# Supplementary material for: Engineered ACE2 receptor therapy overcomes mutational escape of SARS-CoV-2
Source: Nat Commun. 2021 Jun 21;12:3802. doi: 10.1038/s41467-021-24013-y (PMC8217473; doi:10.1038/s41467-021-24013-y)
Supplement: Supplementary file 1 — Supplementary Information [file 41467_2021_24013_MOESM1_ESM.pdf]

Engineered ACE2 receptor therapy overcomes mutational escape of SARS-CoV-2

Supplementary Information

Yusuke Higuchi[1,10], Tatsuya Suzuki[2,10], Takao Arimori[3,10], Nariko Ikemura[1,10], Emiko Mihara[3], Yuhei Kirita[4], Eriko Ohgitani[5], Osam Mazda[5], Daisuke Motooka[6], Shota Nakamura[6], Yusuke Sakai[7], Yumi Itoh[2], Fuminori Sugihara[8], Yoshiharu Matsuura[9], Satoaki Matoba[1], Toru Okamoto[2]*, Junichi Takagi[3]*, Atsushi Hoshino[1]*

[1] Department of Cardiovascular Medicine, Graduate School of Medical Science, Kyoto Prefectural University of Medicine, Kyoto, Japan.
[2] Institute for Advanced Co-Creation Studies, Research Institute for Microbial Diseases, Osaka University, Osaka, Japan.
[3] Laboratory for Protein Synthesis and Expression, Institute for Protein Research, Osaka University, Osaka, Japan.
[4] Department of Nephrology, Graduate School of Medical Science, Kyoto Prefectural University of Medicine, Kyoto, Japan.
[5] Department of Immunology, Graduate School of Medical Science, Kyoto Prefectural University of Medicine, Kyoto, Japan.
[6] Department of Infection Metagenomics, Research Institute for Microbial Diseases, Osaka University, Osaka, Japan.
[7] Department of Veterinary Pathology, Yamaguchi University, Yamaguchi, Japan
[8] The Core Instrumentation Facility, Research Institute for Microbial Diseases, Osaka University, Osaka, Japan.
[9] Department of Molecular Virology, Research Institute for Microbial Diseases, Osaka University, Osaka, Japan.
[10] These authors contributed equally to this work.
*Corresponding author. Email: a-hoshi@koto.kpu-m.ac.jp (A.H.); takagi@protein.osaka-u.ac.jp (J.T.); toru@biken.osaka-u.ac.jp (T.O.)

Items contained in this file:
Supplementary Figures 1-16
Supplementary Table 1-3
References 1-5

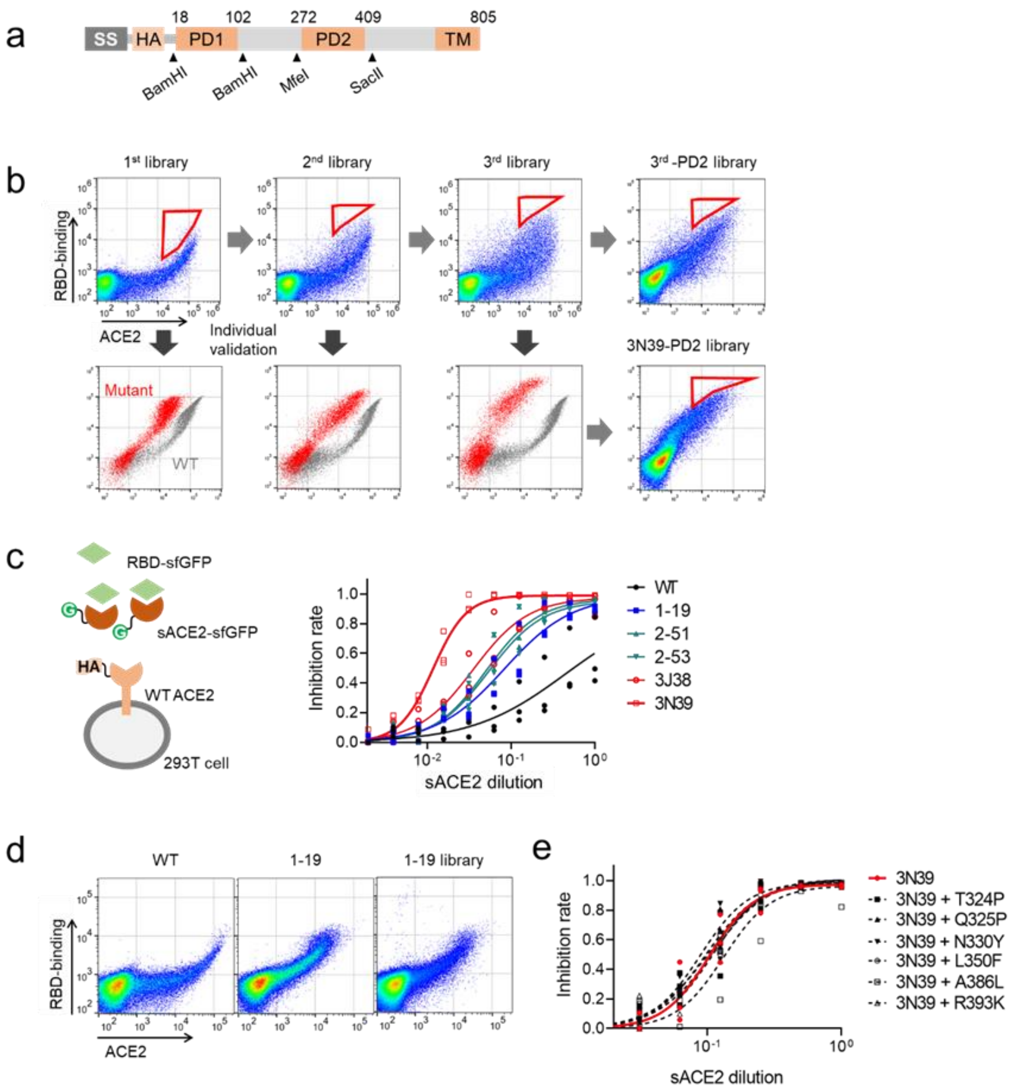

Supplementary Figure 1. Directed evolution to generate high affinity ACE2 in 293T cells. (**a**) Full length ACE2 was optimized to fit screening. Synthetic signal sequence and HA tag were fused to mature ACE2 and restriction sites were introduced by optimizing codon optimization for the mutated fragment replacement. (**b**) PD1 mutagenesis and high affinity selection were performed 3 times and followed by PD2 random mutagenesis. Top 0.05% population were harvested and reconstructed into the backbone plasmid to be verified individually. Cell sorting was conducted by gating on forward scatter (FSC)-H and FSC-A to exclude doublets, followed by gating on Alexa 647 for HA-ACE2 expression and sfGFP for RBD-binding. (**c**) Binding of RBD-sfGFP to wild-type (WT) ACE2 expressing 293T cell in competition with each mutated sACE2-sfGFP. Serial dilution of sACE2-sfGFP was analyzed with the 50-fold diluted RBD-sfGFP in flow cytometry to determine the relative ranking within this experiment (n=3 biological replicate). (**d**) Second mutagenesis based on the top hit, 1-19 mutation in first screening. (**e**) Induction of affinity-enhancing mutation in PD2 from Procko's paper[1] failed to improve the 3N39 capacity of the RBD neutralization. Serial dilution of sACE2-sfGFP was analyzed with the 20-fold diluted RBD-sfGFP in flow cytometry to determine the relative ranking within this experiment. $n = 3$ biological replicate.

a

variant	mutations
3N39	**A25V**, K26E, **K31N**, **E35K**, N64I, **L79F**, N90H
3J113	**K31M**, **E35K**, **Q60R**, S70F, **L79F**, N90D,
3J320	**T20I**, A25V, **H34A**, T78R, **T92Q**, **Q101H**

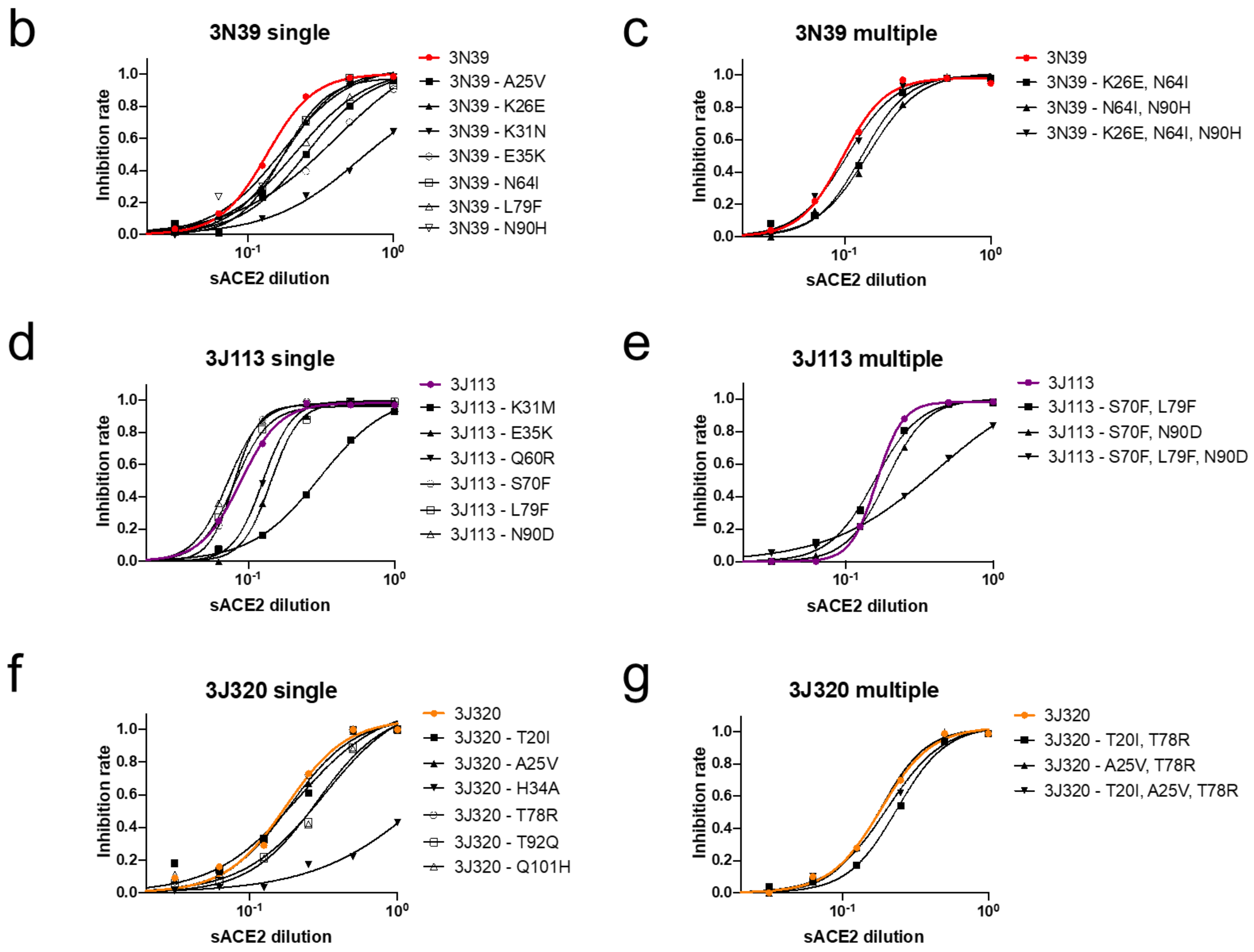

Supplementary Figure 2. The contribution of each mutation in high affinity mutants. Binding of RBD-sfGFP to WT ACE2 expressing 293T cell in the competition with each mutated sACE2-sfGFP. Serial dilution of sACE2-sfGFP was analyzed with 20-fold dilution of the RBD-sfGFP in flow cytometry to determine the relative ranking within each experiment. (**a**) Table of three ACE2 variants and their components. (**b-g**) Each mutation was recovered to wild-type and then analyzed the alteration of neutralization capacity individually in 3N39 (**b**), 3J113 (**d**), and 3J320 (**f**) and in combination with non-essential mutations in 3N39 (**c**), 3J113 (**e**), and 3J320 (**g**). The experiments were independently performed twice and similar results were obtained. One representative data were shown.

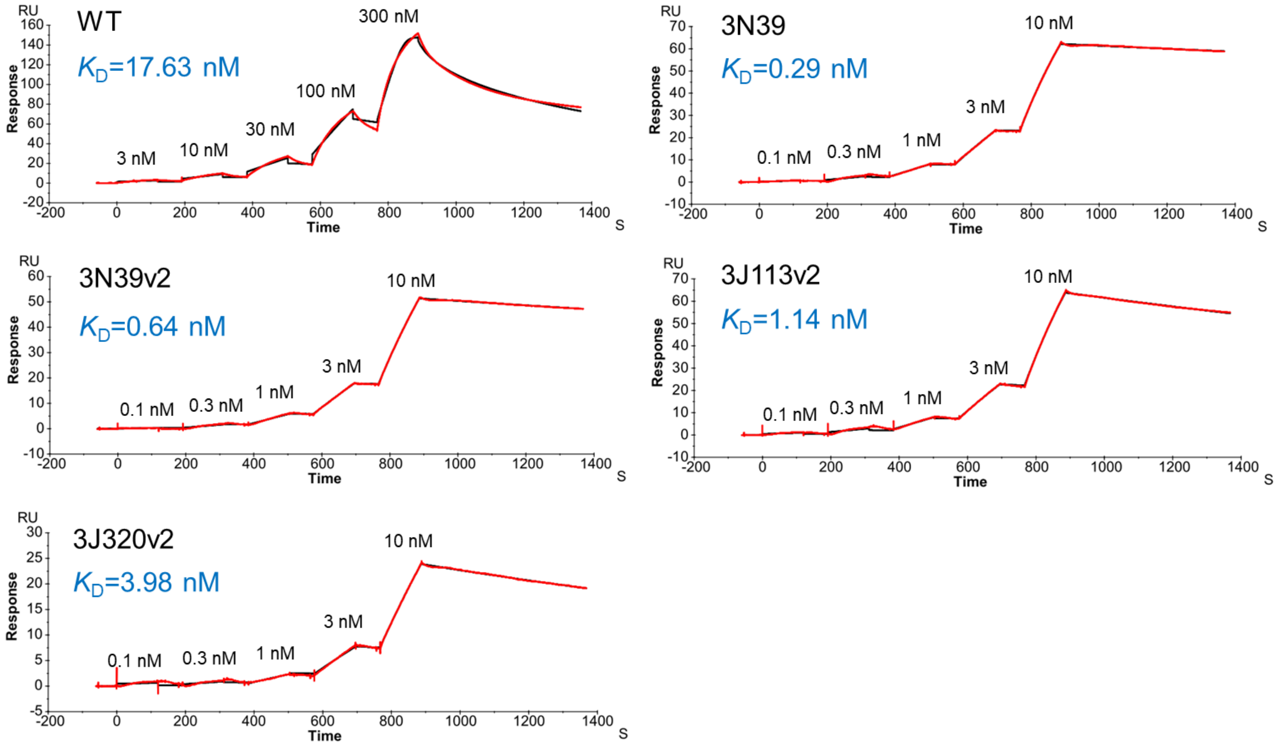

Supplementary Figure 3. Wild-type and mutant sACE2-His bind to the SARS-CoV-2 RBD. Kinetic analysis of sACE2-His binding to RBD-Fc was analyzed by surface plasmon resonance (SPR).

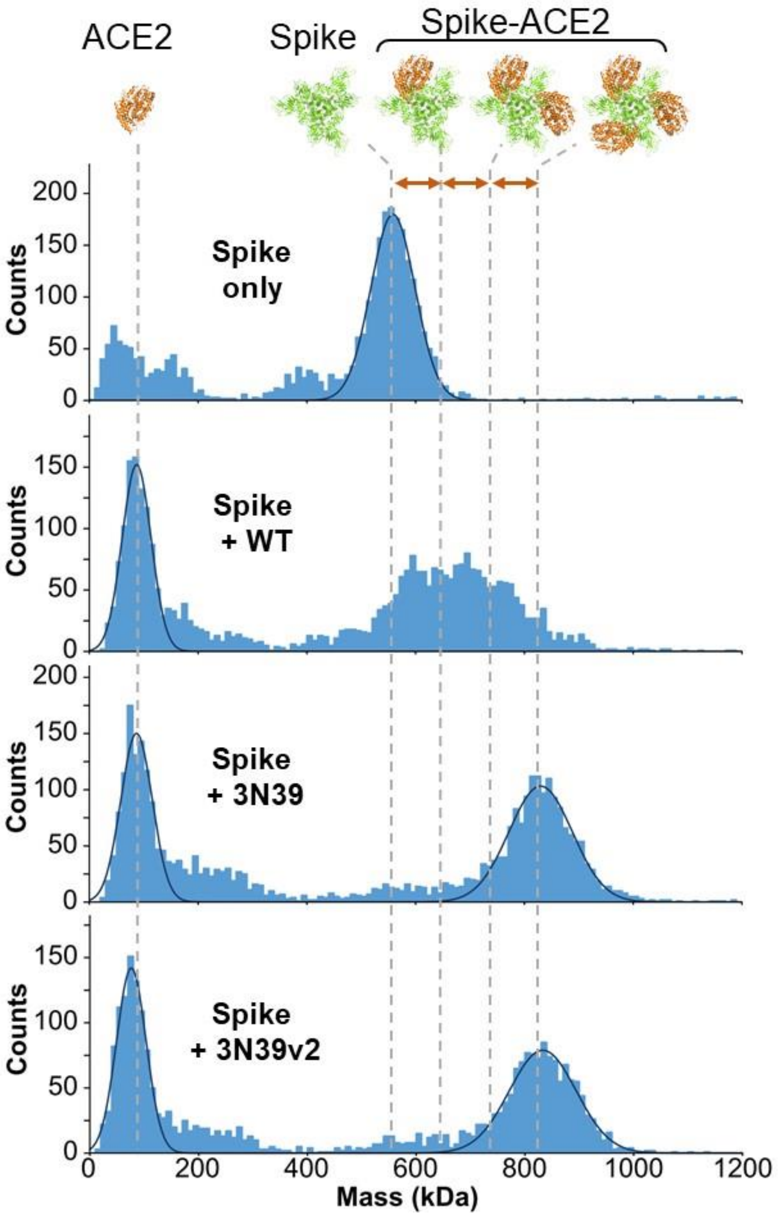

Supplementary Figure 4. Mass photometry histograms of SARS-CoV-2 spike trimer in the absence or presence of ACE2 molecules. An average mass of spike trimer was measured to be ~560 kDa (top panel). All ACE2 molecules added in excess to the mixture samples gave a peak with an average mass of ~90 kDa. The spike protein complexed with WT ACE2 showed a broad mass distribution between 550~850 kDa (the second panel). In contrast, the spike protein complexed with the 3N39 or 3N39v2 showed a monodisperse distribution with an average mass of ~830 kDa (the third and bottom panels), which corresponds to the mass of the spike trimer bound by three ACE2 molecules.

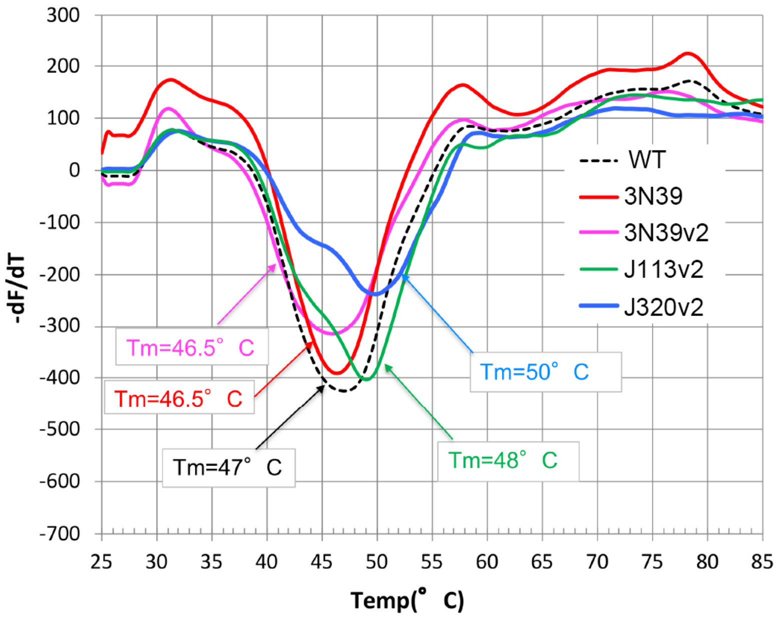

Supplementary Figure 5. Thermostability of ACE2 mutants. Various versions of ACE2-His proteins were subjected to the differential scanning fluorimetry using SYPRO™ Orange as the probe dye. Denaturation curves were replotted for -*d*F/*d*T and the peak temperature was estimated to be the Tm for each mutant.

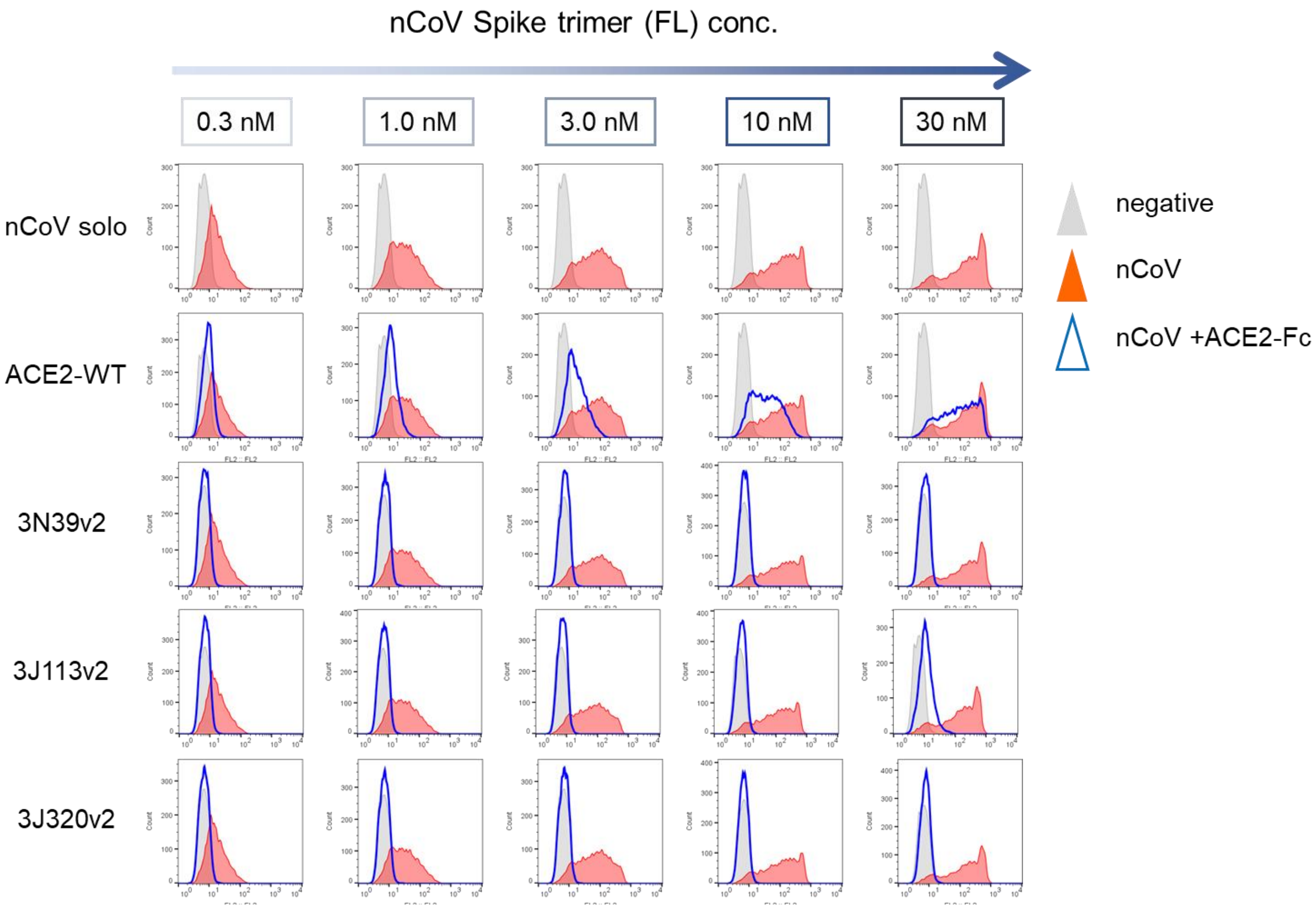

Supplementary Figure 6. Neutralization of spike trimer by ACE2-Fcs. Neutralizing activity of ACE2-Fcs against soluble spike trimer binding to cell-surface ACE2 was evaluated in flow cytometry. Indicated concentration of spike trimer was incubated with 60 μg/ml (~315 nM) ACE2-Fc proteins for 2h and then the mixture was reacted with ACE2-expressing Expi293F cells. Although WT ACE2-Fc can block binding of soluble spike protein to cells when the concentration of the spike was 0.3 nM, it could not outcompete spike at higher concentration. In contrast, complete (for 3N39v2 and 3J320v2) or near-complete (for 3J113v2) inhibition against 30 nM spike protein was achieved with the ACE2 mutants we have isolated, indicating the >100-fold increase in the blocking efficacy from the WT. The experiments were independently performed twice and similar results were obtained. One representative data were shown.

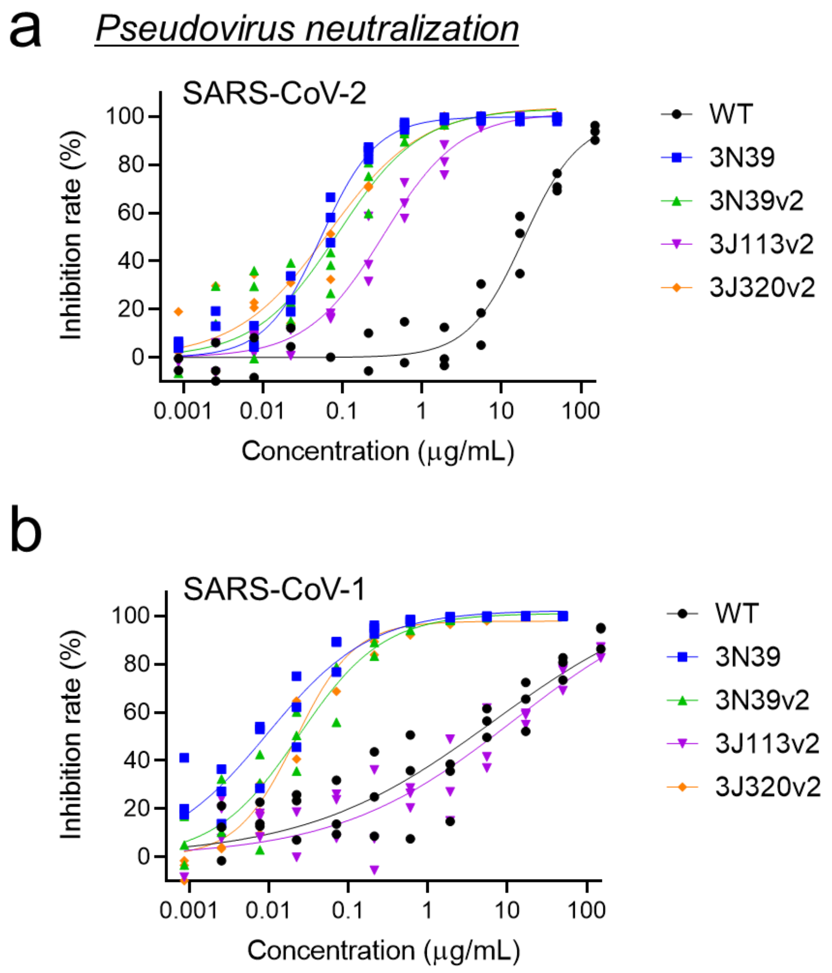

Supplementary Figure 7. High affinity ACE2-Fc neutralized pseudoviruses. (**a**,**b**) Neutralization potency of sACE2-Fc against SARS-CoV-2 (**a**) or SARS-CoV-1 (**b**) pseudotyped lentivirus was measured in 293T/ACE2 cells. *n* = 3 biological replicates.

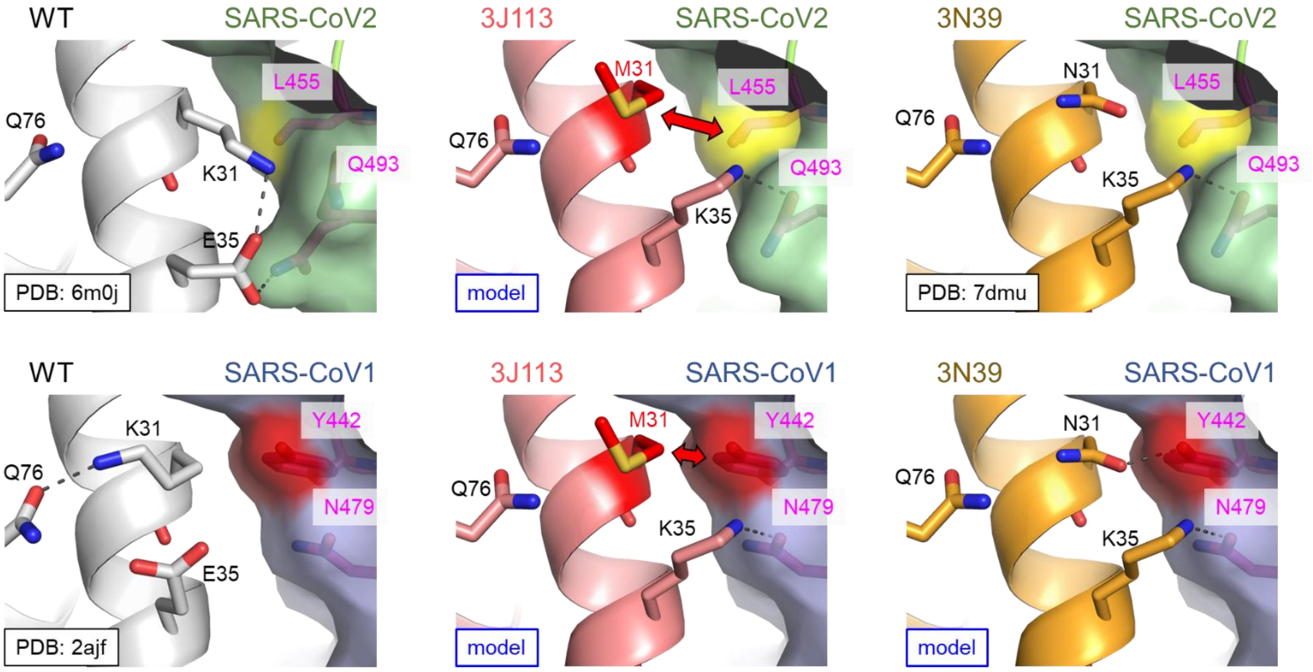

Supplementary Figure 8. The model of 3J113 and SARS-CoV-1 RBD interaction. Structural comparison of the K31M/E35K mutation site in 3J113 (middle panels) with its corresponding site in 3N39 (right panels) and WT (left panels) against L455/Q493 in SARS-CoV-2 RBD (upper panels) and Y442/N479 in SARS-CoV-1 (lower panels). Hydrogen-bonding interactions are indicated by dashed lines.

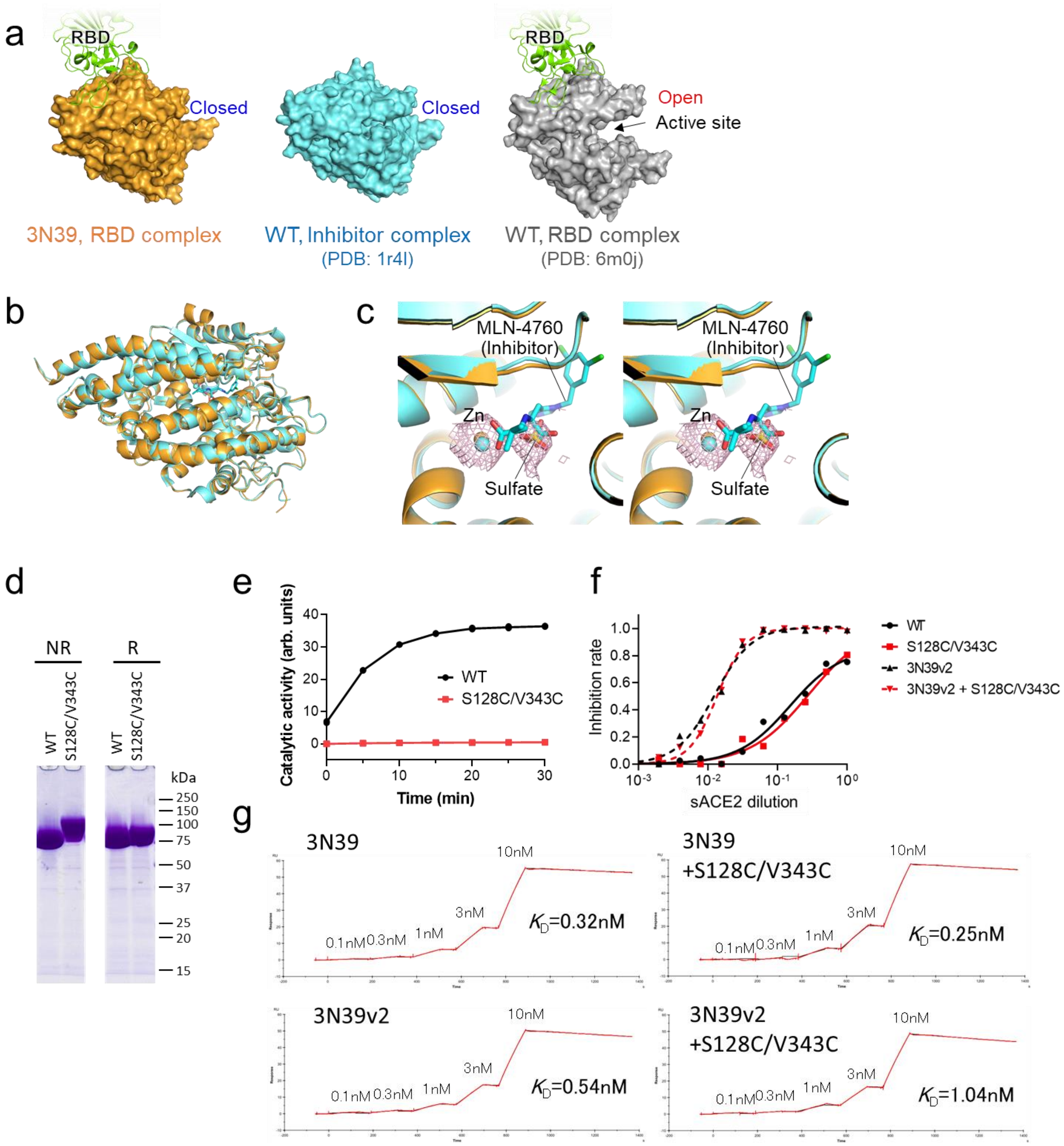

Supplementary Figure 9. Open/closed conformations of ACE2. (**a**) Surface representations of the ACE2 structures. All ACE2 molecules are viewed from the same orientation (side view). The RBD-bound 3N39 (left) and the inhibitor-bound WT (center, 1r4l) adopt a closed conformation, while the RBD-bound WT (right, 6m0j) adopts an open conformation. (**b,c**) Superposition of the RBD-bound 3N39 (orange) and the inhibitor-bound WT (cyan, 1r4l) structures. Overall view and a stereo diagram of the enzymatic active site are provided in (b) and (c), respectively. The active site Zn ions (in both structures) are shown as sphere models, and a sulfate ion (in 3N39) and the inhibitor MLN-4760 (in 1r4l) are shown as stick models. Simulated-annealing F_o-F_c omit map for Zn and sulfate ions contoured

at 3.0 σ is shown in light pink. (**d**) SDS-PAGE analysis of WT and S128C/V343C mutant ACE2-His samples conducted under non-reducing (NR) and reducing (R) conditions. The experiments were independently performed 3 times and similar results were obtained. One representative data were shown. (**e**) Enzyme activity was assayed in the form of soluble ACE2-sfGFP by measuring Mca fluorescence resulting from hydrolysis of Mca-Ala-Pro-Lys(Dnp)-OH. $n = 2$ technical replicates. (**f**) The RBD competitive binding assay was performed in S128C/V343C mutant. Serial dilution of sACE2-sfGFP was analyzed with the 50-fold diluted RBD-sfGFP in flow cytometry to determine the relative ranking within this experiment. The experiments were independently performed twice and similar results were obtained. One representative data were shown. (**g**) Binding kinetics of indicated sACE2-His proteins against RBD-Fc were evaluated by SPR. Shown are K_D values derived from the single-cycle kinetic analysis.

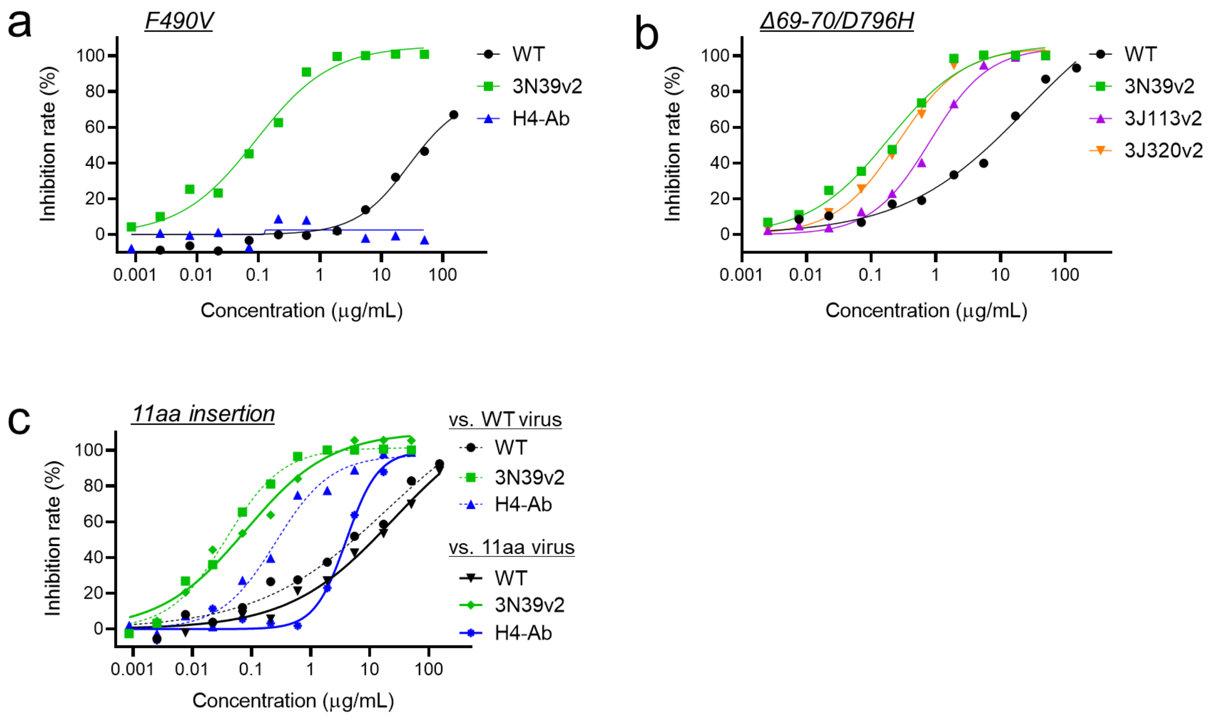

Supplementary Figure 10. The efficient neutralization of ACE2-Fcs against SARS-CoV-2 mutants. (**a**) H4 antibody-resistant mutant, F490V was effectively neutralized by ACE2-Fcs. (**b**) ACE2-Fcs effectively neutralized escape mutants, Δ69-70/D796H that emerged in patient chronically treated with COVID-19 convalescent plasma[2]. (**c**) ACE2-Fcs were effective in the mutant carrying N-linked glycan sequence ($_{248a}$KTRNKSTSRRE$_{248k}$) that was identified in long-term culture[3]. The experiments were independently performed twice and similar results were obtained. One representative data were shown.

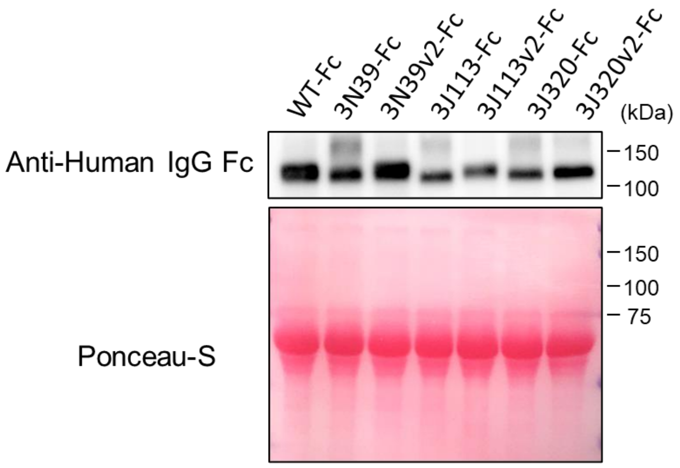

Supplementary Figure 11. The yield of ACE2-Fc proteins. Western blot of cultured medium from each ACE2-Fc transfected 293T cells. The experiments were independently performed 3 times and similar results were obtained. One representative data were shown.

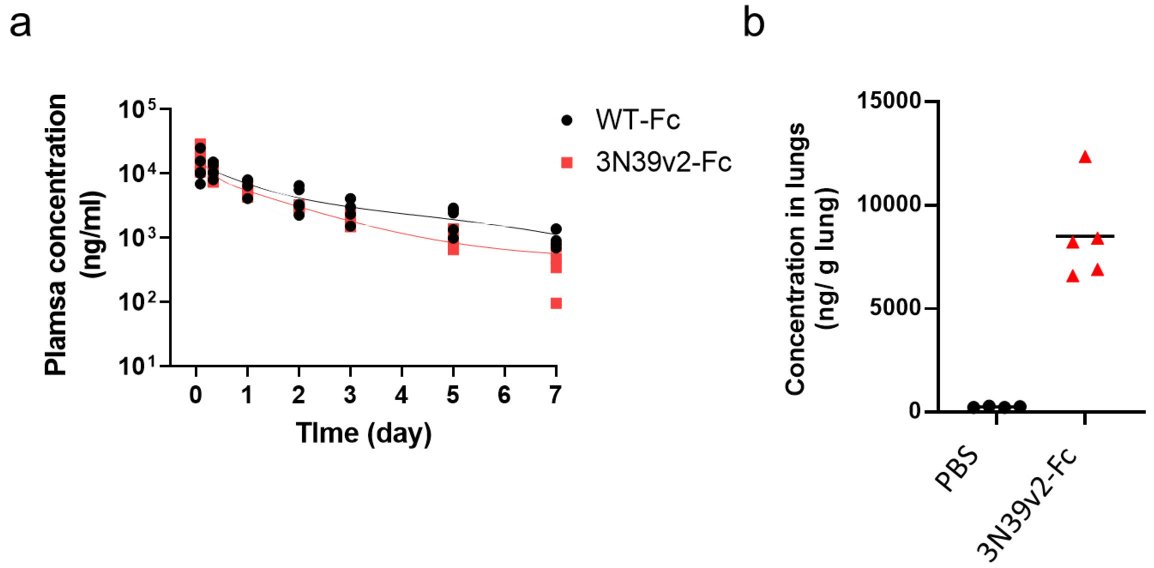

Supplementary Figure 12. Pharmacokinetics of ACE2-Fc after single dose administration. Single dose of WT or 3N39v2-Fc (10mg/kg) was intraperitoneally administered in mice. (**a**) Plasma concentration in the indicated time points was determined by ELISA method. *n* =5 (WT), 4 (3N39v2) from two independent experiments. (**b**) Lung concentration 2 hr after injection was analyzed by ELISA method. *n* = 4 (PBS), 5 (3N39v2).

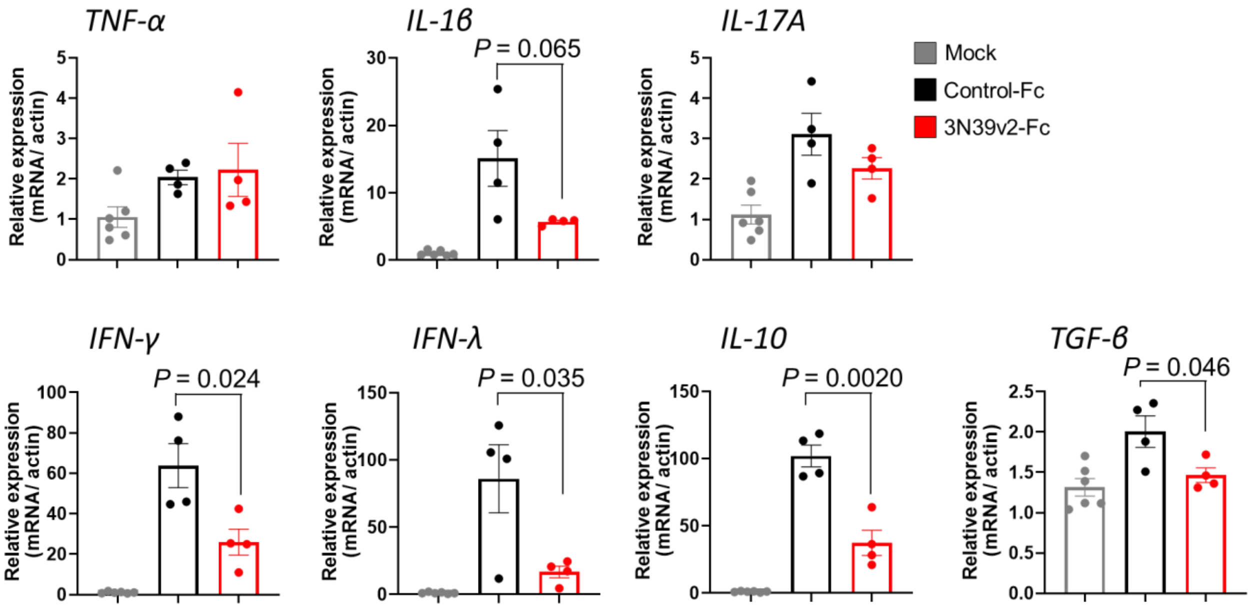

Supplementary Figure 13. Proinflammatory cytokine expression in the lung of COVID-19 hamster. mRNA expression of inflammation-related cytokines in hamster lung lobes. Data are mean ± SEM of n = 6 for mock group, 4 for each treated group. P values by two-sided unpaired t test.

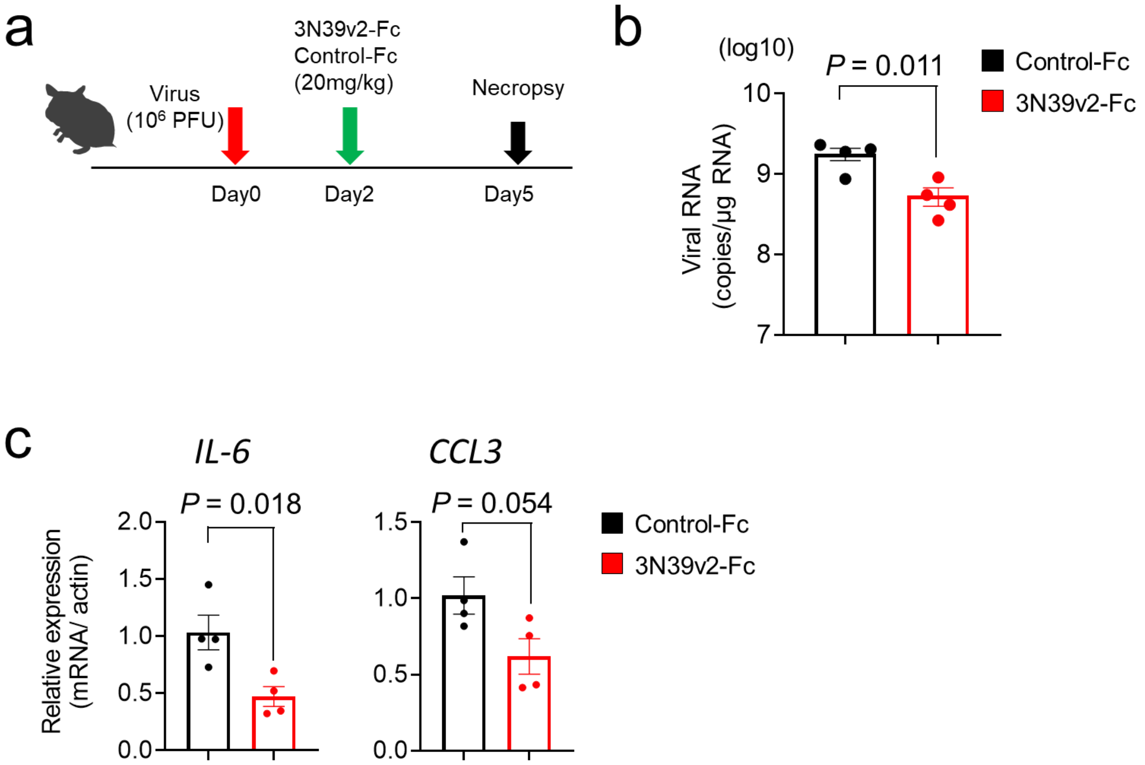

Supplementary Figure. 14. Therapeutic efficiency of 3N39v2-Fc 2 days after virus inoculation (**a**) Schematic overview of the animal experiment. (**b**) Quantification of genomic SARS-CoV-2 RNA as copies per μg of lung tissue transcripts. (**c**) mRNA expression of inflammatory or chemotactic cytokines in hamster lung lobes. Data are mean ± SEM of n = 4 for each treated group. P values by two-sided unpaired t test.

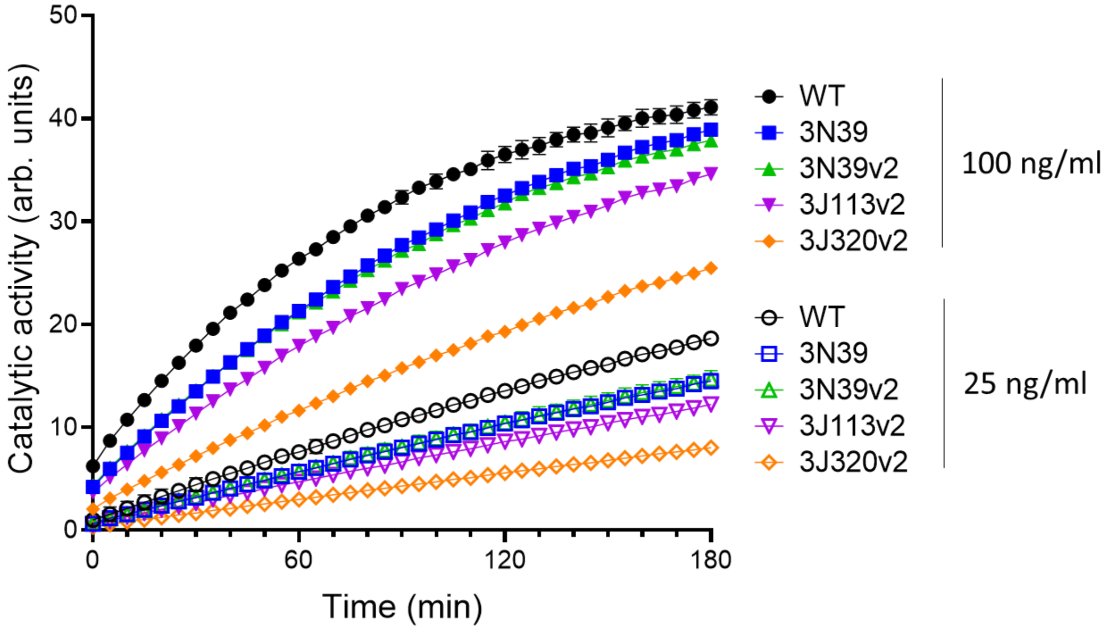

Supplementary Figure. 15. ACE2 catalytic activity of each mutants. Enzyme activity was assayed by measuring Mca fluorescence resulting from hydrolysis of Mca-Ala-Pro-Lys(Dnp)-OH. Fluorescence was recorded every 5 min. Data are mean ± SD of $n = 3$ technical replicates.

Supplementary figure 9d

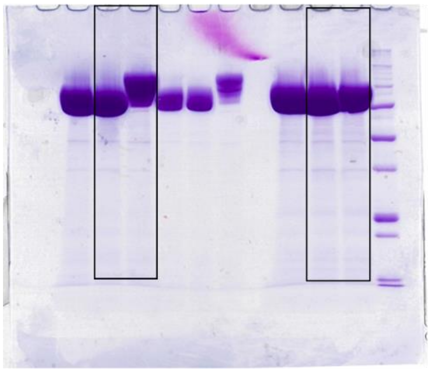

Supplementary figure 11

Anti-Human IgG Fc

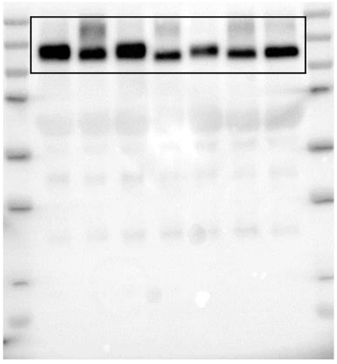

Ponceau-S

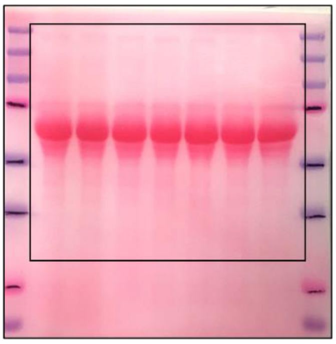

Supplementary Figure 16. Full scans of blot and gel in this study.

Supplementary Table 1. Amino acid sequence and RBD neutralization activity value of validated mutants.

Mutant	Sequence	Value
	20 30 40 50 60 70 80 90 100	
WT	QSTIEEQAKTFLDKFNHEAEDLFYQSSLASWNYNTNITEENVQNMNNAGDKWSAFLKEQSTLAQMYPLQEIQNLTVKLQLQALQQ	-7.24
1-19	QSIIEEQVMTFLDKFNHEAEDLFYQSSLASWKYNTNITEENVQNMNNAGDKWSAFLKEQSTLAQMYPLQEIQNLTVKLQLQALQQ	-2.13
1-19-37	QSIIEEQVMTFLDKFNHEAEDLFYQSSLASWKYNTNITEENVQNMNNAGDKWSAFLKEQSTLAQMYPLQEIQILTVKLQLQALQQ	-5.16
1-19-71	QSIIEEQVMTFLDKFNHEAEDLFYQSSLASWKYNTNITEENVQNMNNAGDKWSAFLKVQSTLAQMYPLQEIQNLAVKLQLQALQQ	-3.03
2-18	QSTIEEEVKTFLDLFNHEAEDLFYQSSLASWNYNTNITEENVQNMNNAGDKWSAFLKEQSSLAQMYPLQEIQTLTVKLQLQALQQ	-2.76
2-45	QSTIEEQAETFLDKFNHEAEDLFYQSSLASWNYNTNITEENVQNMNNAGDKWSAFLKEQSTLAQMYPLQEILNPTVKLQLQALQQ	-1.97
2-51	QSTIEDQVETFLDKFNHEAEDLFYQSSLASWNYNTNITEENVQNMNNAGDKWSAFLKEQSKLAQMYPLQEIQNPTVKLQLQALQQ	-1.78
2-53	HSTIEEQVETFLDKFNHEAEDLFYQSSLASWNYNTNITEENVQNMNNAGDKWSAFLKEQSTLAQMYPLQEIQNLAVMLQLQALQQ	-1.80
3J14	QYTIEDQVETFLDKFNHDAEDLFYQSSLASWIYNTNITEENVENMNNAGDKWSAFLKEQSTLALLYPLQEIQNLQVKLQLQALQQ	-0.92
3J38	KSTTEDQVETFLDKFNHDAEDLFYQSSLASWNYNTNITEENVQNMNNAGDKWSAFLKEQSTLALMYPLQEIQNLQVKLQLLALQQ	-0.85
3J45	QSIIEEQVKTFLEKFSHEAEDLFYQSSLASWNYNTNITEENVQNMNNAGDKWSAFLKEQSTLAQMYPLYEIQNLAVKLQLQALQQ	-2.36
3J80	QSTIEEQVETFLDKFNHEAEDLFYQSSLASWNYNTNITEENVQNMNNAGDEWSAFLKEQSSFAQMYPLQEIQNPTVKLQLQALQQ	-0.16
3J85	LSTIEEQAKTFLDKFNAEAEDLFYQSSLASWNYNTNITEENVQNMINAGDKWSAFLKEQSTLAQMYPLQEIQNLPVKLQLQALQQ	-0.01
3J89	QSTIEEQAKTFLDKFNREAEDLFYQSSLASWNYNTNITEENVQNMNNAGDKWSAFLKEQSTLAQMYPLQEIQDLSVKLQLEALQQ	-2.51
3J109	HSTIEEQVKTFLDMFNHEAEDLFYQSSLASWNYNTNITEENVQDMNNAGDKWFAFLKEQSTLAQMYPLQEIQDLTVKLQLQALQQ	-0.10
3J113	QSTIEEQAKTFLDMFNHKAEDLFYQSSLASWNYNTNITEENVRNMNNAGDKWFAFLKEQSTFAQMYPLQEIQDLTVKLQLQALQQ	0.06
3J120	QSTIEEEAKTFLDKFNHEAEDLFYQSSLASWNYNTNITEENVQNMNNAGDKWSAYLKERSALAQMYPLQEIQNLQVKLQLQALQQ	-0.75
3J121	KYTIEDQVETFLDKFNHEAEDLFYQSSLASWNYNTNITEENVQNMNIAGDKWSAFLKEQSTLAQMYPLQEIQNPTVKLQLQALQQ	-0.19
3J307	HSTTEDQVETFLDKFNHDAEDLFYQSSLASWNYNTNITEENVQYMNNAGDRWSAFLKEQTTFAQMYPLQEIQNLTVMLQLQALQR	-1.10
3J311	QSIIEEQAETFLDKFNHEAEDLFYQSSLASWNYNTNITEENVHNMNYAGDKWSTFLKEQSTVAQMYPLQEIQDLTVKLQLQALQP	-1.34
3J320	QSIIEEQVKTFLDKFNAEAEDLFYQSSLASWNYNTNITEENVQNMNNAGDKWSAFLKEQSRLAQMYPLQEIQNLQVKLQLQALHQ	0.06
3J323	QSTIEDQVETFLDKFNHDAEDLFYQSSLASWNYNTNITEENVQYMNNAGDKWSAFLKEQTALAQMYPLQEIQNLTVKLQLQALQQ	-0.59
3N27	QSTTEEQAETFLDMFNHEAEDLFYQSSLASWNYNTNITKENVQNMNNAGDKWSAFLKEQSTFAQMYPLQEIQDLTVKLQLQALQQ	-3.62
3N39	QSTIEEQVETFLDNFNHKAEDLFYQSSLASWNYNTNITEENVQNMNIAGDKWSAFLKEQSTFAQMYPLQEIQHLTVKLQLQALQQ	0.00
3N68	QSTIEEQAKTFLDKFNHEAEDLFYQSSLASWNYNTNITEEYVQNMNNAGDKWFAFLKEQSTLAQMYPLQEIQKLTVKLQLQALQQ	-5.07
3P4	QSIIEEQVMTFLDNFNHKAEDLFYQSSLASWNYNTNITEENVQNMNIAGDKWSAFLKEQSTFAQMYPLQEIQNLTVKLQLQALQQ	-0.10
3P8	QSTIEEQVETFLDNFNHKAEDLFYQSSLASWNYNTNITEENVLNMNNAWDKWFAFLKEQSSLAQMYPLQEIQNLQVKLQLQALQQ	-1.36

The value of RBD neutralization activity was calculated as -log2 concentration of 50% RBD-sfGFP bound competing relative to 3N39. Contact residues in wild-type ACE2 are retrieved from Shang et al.[4] and Lan et al.[5] (highlighted in blue).

Supplementary Table 2. summary of mutation in the current and previous studies

	PD1																		PD2					
	T20	A25	T27	K31	N33	H34	E35	F40	Q42	Q60	N61	K68	W69	L79	N90	L91	T92	Q101	Q325	N330	H345	H374	H378	A386
3N39v2		A25V		K31N			E35K							L79F										
3J113v2				K31M			E35K			Q60R				L79F										
3J320v2	T20I					H34A											T92Q[†]	Q101H						
Procko-v1						H34A											T92Q[†]		Q325P					A386L
Procko-v2			T27Y											L79T						N330Y				A386L
Procko-v2.1														L79T						N330Y				A386L
Procko-v2.2			T27Y																	N330Y				A386L
Procko-v2.3			T27Y											L79T										A386L
Procko-v2.4			T27Y											L79T						N330Y				
Procko-v3		A25V	T27Y														T92Q[†]		Q325P					A386L
Procko-v4						H34A								L79T						N330Y				A386L
Procko-v5		A25V															T92Q[†]							A386L
Procko-v6			T27Y						Q42L					L79T			T92Q[†]		Q325P	N330Y				A386L
Wells-14						H34V																		
Wells-19				K31F		H34I	E35Q																	
Wells-118						H34V									N90Q[†]							H374N[*]	H378N[*]	
Wells-310		A25V	T27Y			H34A		F40D													H345L[*]			
Wells-311				K31Y									W69V	L79T		L91P[†]					H345L[*]			
Wells-313				K31F	N33D	H34S	E35Q														H345L[*]			
Wells-353			T27A	K31F	N33D	H34S	E35Q				N61D	K68R		L79P							H345L[*]			

[†] mutation losing the N90-glycan

[*] mutation inactivating the ACE2 enzymatic function

Supplementary Table 3. The primers used in this study.

Target gene	Orientation	Sequence
β-actin	Forward	TTGCTGACAGGATGCAGAAG
	Reverse	GTACTTGCGCTCAGGAGGAG
2019-nCoV_N2	Forward	AAATTTTGGGGACCAGGAAC
	Reverse	TGGCAGCTGTGTAGGTCAAC
IL-6	Forward	GGA CAATGACTATGTGTTGTTAGAA
	Reverse	AGGCAAATTTCCCAATTGTATCCAG
CCL3	Forward	GGTCCAAGAGTACGTCGCTG
	Reverse	GAGTTGTGGAGGTGGCAAGG
CCL5	Forward	TCAGCTTGGTTTGGGAGCAA
	Reverse	TGAAGTGCTGGTTTCTTGGGT
CXCL10	Forward	TACGTCGGCCTATGGCTACT
	Reverse	TTGGGGACTCTTGTCACTGG
TNF-α	Forward	TGAGCCATCGTGCCAATG
	Reverse	AGCCCGTCTGCTGGTATCAC
IL-1β	Forward	GGCTGATGCTCCCATTCG
	Reverse	CACGAGGCATTTCTGTTGTTCA
IL-10	Forward	GGTTGCCAAACCTTATCAGAAATG
	Reverse	TTCACCTGTTCCACAGCCTTG
IL-17A	Forward	ATGTCCAAACACTGAGGCCAA
	Reverse	GCGAAGTGGATCTGTTGAGGT
IFN-λ	Forward	CCCACCAGATGCAAAGGATT
	Reverse	CTTGAGCAGCCACTCTTCTATG
IFN-γ	Forward	TGCATCTTGGCTTTGTTGCTC
	Reverse	TCCCCTCCATTCACGACATC
TGF-β	Forward	GGCTACCACGCCAACTTCTG
	Reverse	GAGGGCAAGGACCTTACTGTACTG

References

1. Chan, K.K., *et al.* Engineering human ACE2 to optimize binding to the spike protein of SARS coronavirus 2. *Science* (2020).
2. Kemp, S.A., *et al.* Neutralising antibodies in Spike mediated SARS-CoV-2 adaptation. *medRxiv* (2020).
3. Andreano, E., *et al.* SARS-CoV-2 escape in vitro from a highly neutralizing COVID-19 convalescent plasma. *bioRxiv* (2020).
4. Shang, J., *et al.* Structural basis of receptor recognition by SARS-CoV-2. *Nature* **581**, 221-224 (2020).
5. Lan, J., *et al.* Structure of the SARS-CoV-2 spike receptor-binding domain bound to the ACE2 receptor. *Nature* **581**, 215-220 (2020).
