# Supplementary material for: Engineered ACE2 receptor therapy overcomes mutational escape of SARS-CoV-2
Source: Nat Commun. 2021 Jun 21;12:3802. doi: 10.1038/s41467-021-24013-y (PMC8217473; doi:10.1038/s41467-021-24013-y)
Supplement: Supplementary file 3 — Description of Additional Supplementary Files [file 41467_2021_24013_MOESM3_ESM.pdf]

Description of Additional Supplementary Files

File Name: Supplementary Movie 1
Description: Three-dimensional integrated CT images of the lungs from an uninfected hamster. The movie starts form front view.

File Name: Supplementary Movie 2
Description: Three-dimensional integrated CT images of the lungs from an infected hamster with the control-Fc treatment. Intact area is shown in white invertedly. The movie starts form front view.

File Name: Supplementary Movie 3
Description: Three-dimensional integrated CT images of the lungs from an infected hamster with the 3N39v2-Fc treatment. Intact area is shown in white invertedly. The movie starts form front view.
