# Supplementary material for: Engineered ACE2 receptor therapy overcomes mutational escape of SARS-CoV-2
Source: Nat Commun. 2021 Jun 21;12:3802. doi: 10.1038/s41467-021-24013-y (PMC8217473; doi:10.1038/s41467-021-24013-y)
Supplement: Supplementary file 7 — Reporting Summary [file 41467_2021_24013_MOESM7_ESM.pdf]

nature research

Corresponding author(s): Atsushi Hoshino
Junichi Takagi
Toru Okamoto

Last updated by author(s): May 8, 2021

Reporting Summary

Nature Research wishes to improve the reproducibility of the work that we publish. This form provides structure for consistency and transparency in reporting. For further information on Nature Research policies, see our Editorial Policies and the Editorial Policy Checklist.

Statistics

For all statistical analyses, confirm that the following items are present in the figure legend, table legend, main text, or Methods section.

n/a	Confirmed	
☐	☒	The exact sample size (n) for each experimental group/condition, given as a discrete number and unit of measurement
☐	☒	A statement on whether measurements were taken from distinct samples or whether the same sample was measured repeatedly
☐	☒	The statistical test(s) used AND whether they are one- or two-sided<br>*Only common tests should be described solely by name; describe more complex techniques in the Methods section.*
☒	☐	A description of all covariates tested
☒	☐	A description of any assumptions or corrections, such as tests of normality and adjustment for multiple comparisons
☐	☒	A full description of the statistical parameters including central tendency (e.g. means) or other basic estimates (e.g. regression coefficient) AND variation (e.g. standard deviation) or associated estimates of uncertainty (e.g. confidence intervals)
☐	☒	For null hypothesis testing, the test statistic (e.g. F, t, r) with confidence intervals, effect sizes, degrees of freedom and P value noted<br>*Give P values as exact values whenever suitable.*
☒	☐	For Bayesian analysis, information on the choice of priors and Markov chain Monte Carlo settings
☒	☐	For hierarchical and complex designs, identification of the appropriate level for tests and full reporting of outcomes
☒	☐	Estimates of effect sizes (e.g. Cohen's d, Pearson's r), indicating how they were calculated

Our web collection on statistics for biologists contains articles on many of the points above.

Software and code

Policy information about availability of computer code

Data collection	No software was used
Data analysis	GraphPad Prism 9 for statistical analysis<br>FlowJo v10 for flow cytometry analysis<br>image J for microscopy images analysis and microCT image reconstruction<br>PHENIX 1.14, XDS, COOT 0.8.9, CCP4 v7, MOLPROBITY v.4.5 for crystal structure analysis<br>PyMOL for structural figure preparation<br>Biacore T200 evaluation software v4 for kinetic analysis

For manuscripts utilizing custom algorithms or software that are central to the research but not yet described in published literature, software must be made available to editors and reviewers. We strongly encourage code deposition in a community repository (e.g. GitHub). See the Nature Research guidelines for submitting code & software for further information.

Data

Policy information about availability of data

All manuscripts must include a data availability statement. This statement should provide the following information, where applicable:

- Accession codes, unique identifiers, or web links for publicly available datasets
- A list of figures that have associated raw data
- A description of any restrictions on data availability

Crystallographic coordinates and structure factors for 3N39/SARS-CoV-2 S-RBD complex have been deposited in the Protein Data Bank (PBD) under accession code 7DMU. Other all data generated or analyzed during this study are included in this published article (and its supplementary information files). PBD accession codes for wild-type ACE2/SARS-CoV-2 S-RBD complex and wild-type ACE2/SARS-CoV-1 S-RBD complex are 6m0j and 2ajf, respectively.

Field-specific reporting

Please select the one below that is the best fit for your research. If you are not sure, read the appropriate sections before making your selection.

[x] Life sciences   [ ] Behavioural & social sciences   [ ] Ecological, evolutionary & environmental sciences

For a reference copy of the document with all sections, see nature.com/documents/nr-reporting-summary-flat.pdf

Life sciences study design

All studies must disclose on these points even when the disclosure is negative.

Sample size	No statistical methods were used to predetermine sample size. Sample size was based on experimental feasibility, sample availability, and N necessary to obtain definitive results.
Data exclusions	No data was excluded.
Replication	Neutralization assay against authentic SARS-CoV-2 and pseudovirus were technical 3 replicates and biological 3 replicates, respectively. RBD competition assay was biological 2 or 3 replicates. Hamster infection experiment was performed in n=4 per group.
Randomization	Animals and samples were randomly sorted into experimental groups.
Blinding	The investigators were not blinded to group allocation for data collection and analysis for any experiment performed in this study due to size of the research group with clearance for experimentation under high biocontainment conditions required for work with live SARS-CoV-2.

Reporting for specific materials, systems and methods

We require information from authors about some types of materials, experimental systems and methods used in many studies. Here, indicate whether each material, system or method listed is relevant to your study. If you are not sure if a list item applies to your research, read the appropriate section before selecting a response.

Materials & experimental systems

n/a	Involved in the study	
[ ]	[x]	Antibodies
[ ]	[x]	Eukaryotic cell lines
[x]	[ ]	Palaeontology and archaeology
[ ]	[x]	Animals and other organisms
[x]	[ ]	Human research participants
[x]	[ ]	Clinical data
[x]	[ ]	Dual use research of concern

Methods

n/a	Involved in the study	
[x]	[ ]	ChIP-seq
[ ]	[x]	Flow cytometry
[x]	[ ]	MRI-based neuroimaging

Antibodies

Antibodies used	All antibodies used in the study are listed in Methods.<br>Here we state in order: Antibody name; (Company; Catalogue number; Dilution)<br>anti-HA Alexa 647 (MBL, M180-A64, 1:4000 for FCM)<br>anti-HA Alexa 594 (MBL, M180-A59, 1:1000 for FCM)<br>anti-His Alexa 488 (MBL, D291-A48, 1:1000 for FCM)<br>mouse anti-Spike protein antibody (GeneTex, GTX632604, 1:300 for IHC)<br>goat anti-Human IgG Fc (ThermoFisher, A18817, 1:1000 for WB)<br>goat anti-human IgG1 Fc F(ab')2 (Sigma-Aldrich, I3391, for ELISA)

Validation	All antibodies were validated for specificity and sensitivity using overexpressing cells, infected tissues and negative control samples.

Eukaryotic cell lines

Policy information about cell lines

Cell line source(s)	Lenti-X 293T (Clontech, 632180), VeroE6/TMPRSS2 (National Institutes of Biomedical Innovation, Health and Nutrition, Japan), Expi293F (Thermo Fisher Scientific)
Authentication	Cells were authenticated by morphological appearance.
Mycoplasma contamination	The cell lines were tested for potential mycoplasma contamination and confirmed that they are mycoplasma negative.
Commonly misidentified lines (See ICLAC register)	No commonly misidentified cell lines were used in this study.

Animals and other organisms

Policy information about studies involving animals; ARRIVE guidelines recommended for reporting animal research

Laboratory animals	This study used 4-week-old male Syrian hamsters and 8-week-old male C57BL/6JJcl mice.
Wild animals	This study did not involve wild animals.
Field-collected samples	This study did not involve field-collected samples.
Ethics oversight	All animal experiments with SARS-CoV-2 were performed in biosafety level 3 (ABSL3) facilities at the Research Institute for Microbial Diseases, Osaka University and the study protocol was approved by the Institutional Committee of Laboratory Animal Experimentation of the Research Institute for Microbial Diseases, Osaka University (R02-08-0). Mouse experiments were performed according to procedures approved by Kyoto Prefectural University of Medicine Institutional Animal Care and Use Committees (M2020-63).

Note that full information on the approval of the study protocol must also be provided in the manuscript.

Flow Cytometry

Plots

Confirm that:

- [x] The axis labels state the marker and fluorochrome used (e.g. CD4-FITC).
- [x] The axis scales are clearly visible. Include numbers along axes only for bottom left plot of group (a 'group' is an analysis of identical markers).
- [x] All plots are contour plots with outliers or pseudocolor plots.
- [x] A numerical value for number of cells or percentage (with statistics) is provided.

Methodology

Sample preparation	ACE2 expressing 293T cells were suspended and incubated with RBD-sfGFP and anti-HA alexa 594 or 647.
Instrument	SH800 (SONY), EC800 (SONY), Attune NxT Flow Cytometer (Invitrogen)
Software	FlowJo v10 (FlowJo, LLC)
Cell population abundance	Not confirmed.
Gating strategy	Gating was performed according to sfGFP and alexa594/647 signal strength.

- [x] Tick this box to confirm that a figure exemplifying the gating strategy is provided in the Supplementary Information.
